# Knowledge of Health Professionals on Cold Chain Management and Associated Factors in Ezha District, Gurage Zone, Ethiopia

**DOI:** 10.1155/2019/6937291

**Published:** 2019-06-09

**Authors:** Zeyneba Jemal Yassin, Habtamu Yimer Nega, Behailu Tariku Derseh, Yetnayet Sisay Yehuala, Abel Fekadu Dad

**Affiliations:** ^1^Ethiopian Field Epidemiology and Laboratory Training Program Resident, University of Gondar, Gondar, Ethiopia; ^2^South Wollo Zone, Institute of Public Health Emergency Management Department, Dessie, Ethiopia; ^3^Debre Berhan University, College of Health Sciences, Department of Public Health, Debre Berhan, Ethiopia; ^4^University of Gondar, Institute of Public Health, Department of Health Promotion and Behavioral Sciences, Gondar, Ethiopia; ^5^College of Medicine and Public Health, School of Public Health, Flinders University, Adelaide, Australia; ^6^University of Gondar, Institute of Public Health, Department of Epidemiology Biostatistics, Gondar, Ethiopia

## Abstract

**Background:**

Maintaining quality of vaccines has been one of the main challenges of immunization programs in Africa including Ethiopia, and this could mainly be explained by health professional's knowledge about cold chain management. There are limited studies done in Ethiopia linking the knowledge of health professionals on cold chain management, and that is why we needed to conduct this study.

**Methodology:**

Institution-based cross-sectional study was conducted among all available health professionals in selected health facilities (232 health professionals). Face-to-face interview using a semistructured questionnaire was conducted to collect required information from September to October 2016. Observational checklist was used to spot availability and functionality of refrigerators. Data entry and cleaning was done using Epi Info and exported to SPSS for analysis. A multivariable logistic regression model was fitted to identify factors associated with health professional's knowledge about cold chain management.

**Result:**

The response rate was 92.43%, and 119 (51.3%; 95% CI; 44.9%, 57.6%) health professionals had a satisfactory knowledge about cold chain management. Being trained on immunization program (AOR = 5.1; 95% CI: 2.68, 10.13), having a work experience above six years (AOR = 2.1; 95% CI: 1.8, 4.15), using EPI guidelines (AOR = 2.58; 95% CI: 1.47, 5.57), and being a BSc nurse/health officer (AOR = 2.4; 95% CI: 1.47, 14.4) had got better knowledge on cold chain management.

**Conclusion:**

Health professionals working in the health centers and health posts had low knowledge on cold chain management. Longer work experience, in-service training, and using EPI guideline at work were factors that improved health professionals' knowledge about a cold chain management, which needs to be maintained.

## 1. Background

The Expanded Program on Immunization (EPI) was designed 40 years ago with a set of guidelines for heat-stable but freeze-sensitive vaccines and stable to freezing but heat-labile vaccines [1, 2]. Vaccination is one of the most effective strategies for vaccine-preventable disease when implemented properly across all categories of at-risk population [3, 4]. However, these vaccines are sensitive that slowly lose their potency while stored out of the recommended temperature range [2, 5–8]. Therefore, cold chain management is an essential component of a successful immunization program [4, 9], which maintains the potency of these vaccines [8]. The World Health Organization (WHO) has developed a set of guidelines to properly manage EPI service in its member countries [1, 5, 9].

Vaccines require a conscious effort to maintain the cold chain at different levels [10]. For the EPI to achieve its objective, the cold chain management should be given due emphasis [5], otherwise, cold chain failure will happen and major outbreaks of vaccine-preventable diseases [11] might occur. It is therefore important that health workers should update mechanisms and knowledge to monitor equipment performance and maintain safe temperature in cold rooms, cold boxes, and refrigerators [12].

Inadequate cold chain control of vaccines has been reported in both developed and developing countries [1, 4, 6, 9]. It has also been reported that maintaining quality of vaccines has been one of the main challenges of immunization programs in Africa [2, 5, 7]. A number of studies have been conducted to estimate health professionalsʼ knowledge on cold chain management: a study from Malaysia reported that 78.7% of the health professionals had good knowledge on cold chain management [7]; a cross-sectional study from the North-West region of Cameroon has showed that 37.3% of health workers knew the correct duration of vaccine storage in the health facilities [5]; a study conducted in Lagos, Nigeria, has showed that 95% of health workers had a little knowledge on vaccine vial monitoring (VVM) indicator [9]; and a study in Coastal South India has reported that 60.5% of the health professionals knew how to monitor vaccine temperature and only 22.4% of the health professionals knew about shake test [4]. A study done in the central Ethiopia has stated that 21.6% of health workers knew how to use conditioned ice packs for all types of vaccines during transportation [13].

Factors that affected the level of knowledge on cold chain management have been reported in different studies: gender [5, 7], work experience [5, 13], type and level of profession [13], training on immunization [1, 2, 5, 7, 11], and use of EPI guideline [7, 14]. Moreover, unreliable power sources and limited resources (material, financial, and human) are also factors responsible for the frequent exposure of vaccine to overheating temperature conditions [14].

In Ethiopia, immunization coverage is high while an outbreak, morbidity, and mortality from vaccine-preventable diseases (like measles, pertussis, and neonatal tetanus (NNT)) are still occurring in the form of outbreak in different parts of the country [15]. Studies conducted so far were mainly focused on the coverage of the service while issues with maintaining a cold chain are missing, and factors associated with cold chain management are very scarce and also depend on the local scenarios. Therefore, we were intended to conduct this study to quantify health professionals' knowledge about cold chain management and to identify factors associated with their knowledge. This finding could question the discrepancy between high vaccination coverage, vaccine potency, and occurrence of frequent outbreaks from vaccine-preventable diseases.

## 2. Methods

### 2.1. Study Area

**Figure 1:**
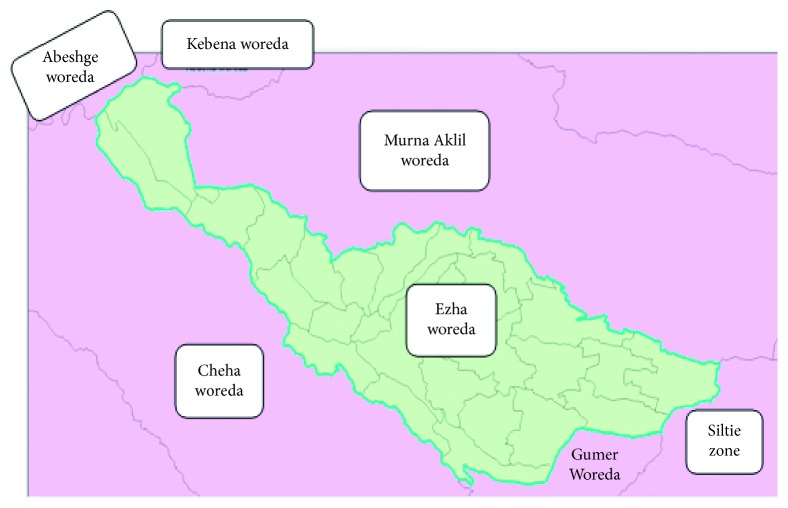
Map of Ezha district (*Source; Ethiopia shape file)*.

The study was conducted in Ezha district governmental health facilities (Figure 1). Ezha is one of the 13 districts and 2 town administration of Gurage zone, which is found in Southern Nation, Nationalities, and Peoples Regional state of Ethiopia. The district has 5 health centers, 31 health posts, and 29 kebeles (the lowest structure in the Ethiopian administrative system). In the district, there are 251 health professions including health extension workers. It is bordered in the south by Gummer woreda, on the west by Cheha woreda, on the north by Kebena and Muhir Na Aklil, and on the southeast by Silte zone. The total estimated population of Ezha woreda in 2016 is 110,021, of this 54,790 (49.8%) were males and 43,769 (50.2%) were females.

### 2.2. Study Design and Population

An institutional-based cross-sectional study was conducted from September to October 2016. All health professionals working in governmental health facilities, 251 health professionals (BSC nurses, Health officer, Midwifery, Diploma nurses, and Health extension workers), and having a responsibility to work on cold chain management were included in the study. Health professionals who were not available during the data collection period were excluded.

### 2.3. Data Collection Tools and Procedures

Face-to-face interview using a semistructured questionnaire was used to collect relevant information regarding sociodemographic characteristics, knowledge-assessing questions on cold chain management, availability of refrigerator, status of refrigerator, and factors affecting the knowledge of health professionals towards cold chain management. The tools were prepared in English and translated into a local language (Amharic) then pretested in health facilities out of the study area (Emdiber Health Center in Cheha district). Secondly, availability and status of the refrigerator, adequacy of cold chain equipment, and logistics of the health facilities were checked by a direct observation. Three newly graduated health officers were recruited and trained, and they collected the data. The principal investigator supervised and managed the overall data collection process.

### 2.4. Main Outcome Variable Measurement

Knowledge towards a cold chain management: a sixteen-item questionnaire was used to assess health professionals' knowledge. Participants' total knowledge score was obtained after adding each response (if they provided the correct answer, they got 1 or else 0 points), and health professionals who scored 50% and below were labeled as having unsatisfactory knowledge, while those who scored above 50% were labeled as having a satisfactory knowledge.

### 2.5. Data Processing and Analysis

Data were entered and cleaned by using Epi Info software, thereafter exported to SPSS Version 20 for further analysis. Exploratory data analysis was conducted to describe the nature of the data. A multiple logistic regression model was fitted to identify predictors of having a satisfactory knowledge on cold chain management. Crude and adjusted odds ratio with its 95% confidence interval was determined as a measure of association, and a *p* value <0.05 was used to portrait a level of significance.

### 2.6. Ethical Considerations

The ethical approval of this study was secured from the Review Board of the University of Gondar, and permission to conduct the study was obtained from Ezha district health office. Oral informed consent and parental consent for study participants with age less than 18 years was obtained from all study participants, and obtained information was also kept confidential through using codes of the study participants, and all the information obtained was locked in a cupboard.

## 3. Results

Assessment of knowledge on cold chain management was made for 232 health professionals (92.4% of the planned sample). The majority of the study participants (88, 37.9%) were nurses by profession. The mean age of the respondents was 27 ± 4.6 (SD), and the majority of them were 169 (72.8%) in the age category of 25–34 years. Out of the total respondents, 133 (57.3%) of them were females. Excess number of health professionals, 155 (66.8%), were not trained on immunization package (Table 1).

### 3.1. Infrastructure and Cold Chain Equipment

Eight health facilities (22.8%) had a refrigerator, while the remaining facilities transport vaccines from nearby health facilities that had a functional refrigerator. Five (62.5%) health facilities had car/motorbike for transportation of vaccines in case of refrigerator power failure. Out of eight facilities, seven of them had solar functioning refrigerators. Seven (87.5%) facilities were permanently assigned personnel that follow cold chain during the working hours (Table 2).

### 3.2. Cold Chain Condition of the Health Facilities

Out of eight health facilities that had a functional refrigerators, seven (87.5%) had functional fridge tag. On the day of data collection, out of 7 functional fridge tags, 6 (85.7%) showed temperature readings within the standard range (2°C–8°C). Six out of 7 (85.7%) functional fridges had two updated recordings every day. During the data collection period, laboratory reagents and maternity medicines were placed with vaccines in 2 of the 8 (25%) health facilities (Table 3).

### 3.3. Knowledge of Health Professionals on Cold Chain Management

One hundred sixty-five (71.1%) and 194 (83.6%) health workers correctly mentioned the recommended range of temperature (2°C–8°C) for vaccine storage and the frequency of temperature recordings, respectively. Respondents were assessed on their ability to mention the proper compartment placement in a vertical (upright) or chest type of refrigerators. Of those asked, 164 (70.1%) correctly replied for polio vaccine (OPV), 84 (36%) for tetanus toxoid (TT), 182 (78.4%) for pentavalent vaccines (DTPHBHib), 151 (65.1%) for measles, 181 (78%) for pneumococcal conjugate vaccines (PCV), and 152 (65.5%) for ROTA vaccines. Overall, 119 (51.3%; 95% CI; 44.9%, 57.6%) of health professionals had a satisfactory knowledge on cold chain management.

### 3.4. Factors Associated with Satisfactory Knowledge on Cold Chain Management

After controlling the confounding effect of age, place of work, and sex of the respondents, type of professions, years of experience, use of EPI guideline, and being trained on cold chain management were statistically associated with having a satisfactory knowledge. Hence, health officers and nurses (BSc) were 2.40 (AOR = 2.4; 95% CI: 1.47–14.4) times more knowledgeable on cold chain management. Health workers who had a working experience of six and more years were 2.10 (AOR = 2.1; 95% CI: 1.8–4.15) times more knowledgeable as compared to those who had less than two years. Health professionals who use EPI guidelines were 2.58 (AOR = 2.8; 95% CI: 1.47–5.57) times more likely to have a satisfactory knowledge on cold chain management as compared to those who had no experience of using the guidelines. Moreover, workers who had a training on immunization were 5.10 (AOR = 5.1; 95% CI: 2.68–10.13.) times more knowledgeable on cold chain management (Table 4).

## 4. Discussion

This study provides insight into health professionals' knowledge on cold chain management and its related factors among health professionals at governmental health facilities in Ezha district. This study showed that 51.3% of health professionals had a satisfactory knowledge on cold chain management. This figure is nearly consistent with a study done in central Ethiopia in which 56% of health professionals had a satisfactory knowledge on cold chain management [13]. However, it is much lesser than a study conducted in Malaysia in 2013 that reported 78.2% [7]. This discrepancy might be due to a difference in staff motivation and study participants' qualification as a study in Malaysia included only medical doctors. In this study, about 71.1% of the respondents knew the recommended range of temperature for vaccine storage. This finding is consistent with a study done in Western India in 2013 in which 80% of professionals knew the recommended range of vaccine storage [3] but this result is higher than a study conducted in Cameroon in 2015 [5], and this discrepancy might be due to a geographical and sociocultural differences in the studies.

Sixty-five percent of the health professionals knew the proper compartment of measles vaccine. This finding is nearly similar with a study done in the North-Western region of Cameroon in 2015 in which 62.3% of health professional knew the exact compartment of measles vaccine [5]. However, it is slightly different from a study done in central Ethiopia in 2012 in which 71.6% of health professionals knew the exact compartment of measles vaccine [13]. This discrepancy might be due to an absence or insufficient training, work load, or staff turnover.

The finding of this study showed that 14.6% of health workers knew the purpose for application of a shake test for detecting flocculation of TT vaccine. This result is much less than that of a study conducted in western India [3] and in central Ethiopia [13]. More than one-third (44.4%) of health professionals were able to list a vaccines that are most sensitive to heat. This figure is consistent with a study done in central Ethiopia [13] but much lesser than a study conducted in Malaysia in 2013 [7]. One-third (33.2%) of health professionals knew the vaccines that are most sensitive to cold, and this finding is higher than a study done in central Ethiopia [13] but lesser than a study conducted in Malaysia [7].

In the present study, less than one-third (22.8%) of health facilities had functional refrigerator. This finding is nearly similar to a study done in central Ethiopia in 2016 in which 19% of the facilities had a functional refrigerator [13], but it is lesser than a study conducted in the North-West Region of Cameroon in which 95.1% of health facilities had a functional refrigerator with a working thermometer [5]. This discrepancy might be due to a difference in the socioeconomic characteristics between the countries and the purpose of the study as a study done in the North-Western Cameroon was conducted in a pilot site.

On the day of data collection, 85.7% of the refrigerator thermometer readings showed within a standard range (2°C–8°C). This figure is consistent with a study conducted in Saudi Arabia [4] but higher than a study conducted in central Ethiopia [13]. However, on the other side, sometimes a short span of temperature recordings does not certify a cold chain quality as reported by a study in Burkina Faso [16]. In this study, we observed that 25% of facilities who had a refrigerator kept ergometrine and oxytocin injection with vaccines. This finding was higher than that of a study conducted in central Ethiopia in 2012 [13], but it was higher than that of the study in South India [4]. This might be due to lack of motivation, supervision, negligence of the professionals, and a poor knowledge towards the effect of repeatedly opening the fridge on vaccine potency. Different studies outlined the problem of keeping vaccines together with laboratory specimens, drugs, food, or drinks [7, 9].

This study showed health workers who trained about cold chain management had better knowledge than their untrained friends. This result is consistent with a study done in Malaysia [7]. In addition, BSc nurses or health officers were 2.4 times more knowledgeable about cold chain management than the health extension workers. Similar result was reported in a study done in central Ethiopia in 2012 [13] where the relationship between level of professions and knowledge on cold chain management was statistically significant (*p* < 0.05). Use of guideline by health workers believed to improve the cold chain management as this increases their knowledge on the area as reported by a study in the North-West Region of Cameroon and Malaysia [7, 14]. Consistently, our study identified that health professionals who use EPI guidelines had a better knowledge on cold chain management than those professionals who did not use.

The finding of this study also showed that health workers whose working experience was more than six years knew more about a cold chain management than those who had a shorter experience, and a similar result was reported in a study done in central Ethiopia and Malaysia [7, 13]. This showed experience by itself is a school by which an individual could learn from himself or from his staff through his daily activities. A cross-sectional nature of this study might make it harder to see a temporal relationship and its limited scope might hinder the generalization of the study finding to wider community. However, as information on a cold chain management is very scarce in Ethiopia, this finding might create a question why there are outbreaks in areas where high vaccine coverage is attained.

## 5. Conclusion

In conclusion, health professionals' knowledge on cold chain management was low. We also found that studies conducted on the same or similar topic are very limited in Ethiopia. In addition to that, health professionals had limited knowledge on vaccine shake test and they were also hardly mentioned vaccine sensitivity to heat, cold, and light. Having longer work experience, better professional qualification, being trained on immunization, and use of guideline were the factors that increased the odds of having a better knowledge on cold chain management. Therefore, in-service training shall be given for health professionals, particularly on proper placement of vaccines and the application of shake or VVM test. In addition, monitoring and evaluation of EPI shall be strengthened by providing an EPI guideline for all health facilities. Finally, a direct-drive solar refrigerator shall be distributed for those health facilities which do not have electricity.

## Figures and Tables

**Table 1:** Percentage distribution of respondents by sociodemographic characteristics among health professionals working in Ezha district, Gurage zone, Ethiopia, 2016.

Variables	Category	Frequency	Percent (%)
Respondent place of work (*n* = 232)	Urban	41	17.7
	Rural	191	82.3

Responsibilities (*n* = 232)	HC professionals	177	76.3
	HP professionals	55	23.7

Age (*n* = 232)	15–24 years	51	22
	25–34 years	169	72.8
	≥35 years	12	5.1

Sex (*n* = 232)	Female	133	57.3

Type of profession (*n* = 232)	Health extension workers	55	23.7
	Diploma nurses	103	44.4
	BSC nurse/health officer	48	20.7
	Midwifery	26	11.2

Work experience (*n* = 232)	6 months–2 years	44	19
	2 years–4 years	109	47
	4 years–6 years	38	16.4
	≥6 years	41	17.7

Received training on immunization (*n* = 232)	Yes	77	33.2
	No	155	66.8

Days of stay on immunization training (*n* = 77)	Less than 3 days	20	26
	3–5 days	45	58.4
	≥6 days	12	15.6

Received training on fridge maintenance (*n* = 232)	Yes	2	0.9
	No	230	99.1

**Table 2:** Infrastructure and cold chain equipment/resource availability in Ezha district, Gurage zone, Ethiopia, 2016.

Characteristics	Frequency	
	Yes (%)	No (%)
Availability of refrigerator in the health facility (*n* = 35)	8 (22.8)	27 (77.2)
Availability of functional refrigerator (*n* = 35)	8 (22.8)	27 (77.2)
Availability of functional generator/solar (*n* = 8)	7 (87.5)	1 (12.5)
Availability of functional car/motorbike in the facilities to use in case of refrigerator failure (*n* = 8)	5 (62.5)	3 (37.5)
Availability of trained personnel for minor fridge maintenance (*n* = 8)	2 (25)	6 (75)
Availability of spare parts for minor fridge maintenance (*n* = 8)	0	8 (100)
Availability of permanently assigned personnel for cold chain management (*n* = 8)	7 (87.5)	1 (12.5)
Availability of personnel assigned during holidays/weekend for cold chain follow up (*n* = 8)	4 (50)	4 (50)
Availability of kerosene for refrigerator (*n* = 8)	0	8 (100)
Using EPI guidelines or manual (*n* = 35)	4 (11.4)	31 (88.6)

**Table 3:** Cold chain status in Ezha district, Gurage zone, Ethiopia, 2016.

Characteristics	Frequency	%
Functional fridge tag availability (*n* = 8)	7	87.5
The refrigerator showed temperature readings within the standard range (2°C–8°C) (*n* = 7)	6	85.7
In the last 1 month the fridge tag alarmed (*n* = 7)	5	71.4
Action taken after the fridge tag is alarmed (*n* = 7)	5	71.4
Daily temperature recording chart availability (*n* = 7)	7	100
Temperature recorded twice daily (*n* = 7)	6	85.7
Vaccine arrangement as recommended standard (*n* = 8)	8	100
Availability of other drugs with EPI vaccines (*n* = 8)	2	25

**Table 4:** Bivariable and multivariable analysis of knowledge on cold chain management among health professionals in Ezha district, Gurage zone, Ethiopia, 2016.

Variables	Level of knowledge		COR (95%, CI)	AOR (95%, CI)
	Satisfactory	Unsatisfactory		
*Respondent place of work*				
Urban	24	17	0.79 (0.27, 2.38)	
Rural	95	96	1	

*Sex*				
Male	59	43	1.69 (0.67, 4.57)	
Female	60	70	1	

*Type of profession*				
HEW	13	42	1	1
Diploma nurses	66	37	1.43 (0.34, 5.6)	1.40 (0.50, 4.06)
BSC/HO	32	16	1.67 (0.65, 4.23)	2.40 (1.47, 14.4)^*∗*^
Midwifery	8	18	2.10 (0.68, 5.87)	1.20 (0.80, 4.52)

*Work experience*				
6 months–2 yrs	20	24	1	1
2–4 years	58	51	2.60 (1.83, 6.40)	0.70 (0.32, 1.82)
4–6 years	25	13	1.20 (3.60, 8.00)	0.50 (0.27, 1.17)
≥6 years	16	25	3.00 (0.59, 16.12)	2.10 (1.80, 4.15)^*∗*^

*Receiving training on EPI*				
Yes	59	18	2.80 (1.89, 6.76)	5.19 (2.68, 10.13)^*∗*^
No	60	95	1	1

*Availability of functional refrigerators*				
Yes	87	75	2.70 (1.38, 3.70)	
No	32	38	1	

*Used guidelines*				
Yes	80	50	0.61 (0.23, 1.61)	2.58 (1.47, 4.57)^*∗*^
No	39	63	1	1

## Data Availability

The datasets used and/or analyzed during the current study are available in the manuscript.

## Abbreviation

CC: Cold chain
AOR: Adjusted odds ratio
COR: Crude odds ratio
EPI: Expanded Program on Immunization
NNT: Neonatal tetanus
OPV: Oral polio virus
PCV: Pneumococcal conjugated vaccine
TT: Tetanus toxoid
VVM: Vaccine vial monitoring
WHO: World Health Organization.
